# African Plant-Based Natural Products with Antivirulence Activities to the Rescue of Antibiotics

**DOI:** 10.3390/antibiotics9110830

**Published:** 2020-11-19

**Authors:** Christian Emmanuel Mahavy, Pierre Duez, Mondher ElJaziri, Tsiry Rasamiravaka

**Affiliations:** 1 Laboratory of Biotechnology and Microbiology, University of Antananarivo, BP 906 Antananarivo 101, Madagascar; mitsinjomahavy@gmail.com; 2 Laboratory of Plant Biotechnology, Université Libre de Bruxelles, B-1050 Brussels, Belgium; jaziri@ulb.ac.be; 3 Unit of Therapeutic Chemistry and Pharmacognosy, University of Mons, 7000 Mons, Belgium; pierre.duez@umons.ac.be

**Keywords:** antivirulence, African plants, biofilm, *Escherichia*, natural compounds, *Pseudomonas*, quorum sensing, *Ralstonia*, *Staphylococcus*

## Abstract

The worldwide emergence of antibiotic-resistant bacteria and the thread of widespread superbug infections have led researchers to constantly look for novel effective antimicrobial agents. Within the past two decades, there has been an increase in studies attempting to discover molecules with innovative properties against pathogenic bacteria, notably by disrupting mechanisms of bacterial virulence and/or biofilm formation which are both regulated by the cell-to-cell communication mechanism called ‘quorum sensing’ (QS). Certainly, targeting the virulence of bacteria and their capacity to form biofilms, without affecting their viability, may contribute to reduce their pathogenicity, allowing sufficient time for an immune response to infection and a reduction in the use of antibiotics. African plants, through their huge biodiversity, present a considerable reservoir of secondary metabolites with a very broad spectrum of biological activities, a potential source of natural products targeting such non-microbicidal mechanisms. The present paper aims to provide an overview on two main aspects: (i) succinct presentation of bacterial virulence and biofilm formation as well as their entanglement through QS mechanisms and (ii) detailed reports on African plant extracts and isolated compounds with antivirulence properties against particular pathogenic bacteria.

## 1. Introduction

Antimicrobial resistance, increasingly observed within a wide range of pathogenic bacteria, has become a worldwide threat to public health [1,2]. Over time, bacteria adapt to the drugs that are designed to kill them, evolving or selecting resistance mechanisms to ensure survival. The resistance of bacteria to antibiotics is a naturally occurring phenomenon, supposedly progressive over contact with antibiotics. However, the misuse and abuse of antibiotics led to a strong and rapid selective pressure, leading to an uncontrolled widespread development of antibiotic-resistant bacteria [3,4]. Beyond resistance to antibiotics, the ability of bacteria to develop effective biofilms represents one of the major obstacles in the fight against bacterial infections. Indeed, while planktonic lifestyle allows bacteria to easily diffuse in diverse environments, their biofilm lifestyle allows efficient colonization of biotic and abiotic surfaces and protection from environmental aggression [5].

Undoubtedly, whenever new antimicrobial compounds would be discovered, their use will result in selective pressures, probably leading targeted bacteria to develop a resistance to these agents. This likely outcome stirs researchers to consider other strategies, notably based on the search for original compounds that impair virulence expression mechanisms and/or biofilm formation without affecting bacterial viability [6,7]. Striking such targets will likely impact invasion capabilities and aggressiveness of bacteria, as well as their ability to build protective barriers against host immune defenses or antibiotics; all the while, selective pressure would be minimized [8], most probably preventing or slowing down the apparition and spread of resistances.

The expression of bacterial virulence factors is generally coordinated by quorum sensing (QS) mechanisms, a cell-to-cell communication system that allows bacteria to detect their population density by producing and perceiving diffusible signal molecules that synchronize common behaviors [6]. Depending on bacteria species, QS regulates the production of virulence factors, motilities, and/or biofilm formation. Thus, the disruption of QS signaling, also termed quorum quenching (QQ), appears as interesting adjuvant strategies in the fight against bacterial infections [9].

Over millions of years of co-evolution, plants accumulated highly diverse secondary metabolites (so-called ‘natural products’), developing means of surviving in hostile environments that combine herbivorous insects and pathogenic bacteria, fungi, and viruses [10]. Given the huge diversity of flora and ecosystems in the world, the plants likely represent significant sources of innovative compounds with antivirulence properties. Indeed, several studies have already reported natural compounds, mainly isolated from plants, and synthetic compounds interfering with bacterial virulence [5,11]. For instance, ajoene, an allyl sulfide isolated from garlic (*Allium sativum* L., Liliaceae) and baicalin, a flavone glycoside isolated from Huangqin (*Scutellaria baicalensis* Georgi, Lamiaceae) have been reported to inhibit both virulence factors production and biofilm formation in *P. aeruginosa* through QQ pathways [12,13].

From an estimated African biodiversity of ~45,000 plant species, only 5000 have documented medicinal use [14]. The list of drugs provided by the African flora appears quite short (less than 100 active compounds) [15] compared with those from other traditional medical systems such as Traditional Chinese Medicine (more than 2000 active compounds from Chinese herbal) [16], suggesting an unrivalled opportunity for the discovery of new drugs. The present review aims to report on African plants raw extracts and isolated natural compounds with antivirulence properties against particular pathogenic bacteria. As a prerequisite, and to better document these potential antivirulence activities, the first sections of this paper provide an overview of bacterial virulence mechanisms, biofilm formation processes, and their regulation via QS mechanisms. The possible contribution of such antivirulence agents for an efficient control of infective bacteria is also discussed. The literature has been collected from electronic databases such as PubMed, Scopus, and ScienceDirect until August 2020, using relevant keywords including “african medicinal plants, extracts, natural products, virulence, quorum quenching, quorum sensing, biofilm”, and their combination.

## 2. Bacterial Virulence

**Figure 1:**
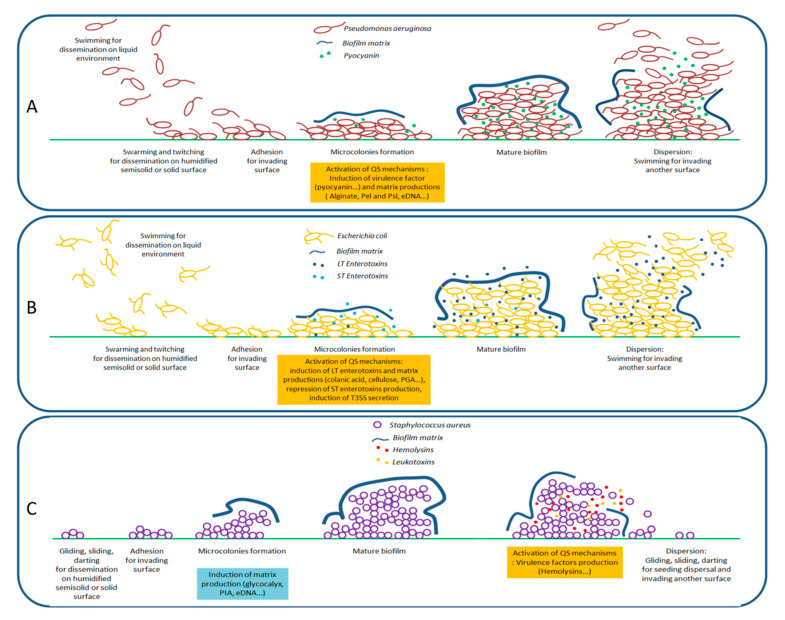
Schematic representation of bacterial invasion processes (**A**) *P. aeruginosa*, (**B**) *E. coli*, and (**C**) *S. aureus*. Bacterial dissemination is done thanks to active and/or passive motilities (swimming, swarming, twitching, gliding, sliding, and darting). In presence of appropriate surfaces and environment, the invasion is initiated by adhesion steps and microcolony formation which lead to the development of mature biofilms in four major steps (adhesion, microcolonies development, biofilm maturation, and dispersion). Under modulation by QS, the bacterial community deploys an arsenal of virulence factors (pyocyanin, heat labile (LT) and heat stable (ST)-enterotoxin, hemolysins, and leukotoxins) that undermine nearby cells (including immune defense cells) and stimulate the production of exogenous biofilm matrix for protection purposes.

Infections by bacteria are mainly related to their ability to invade and disseminate through their hosts by using different types of motility, by releasing a myriad of virulence factors, by building structured biofilm which lead to host cell and tissue damages, but also to evade immune defense systems [17,18]. The present section will briefly discuss these bacterial virulence and invasion processes, schematically summarized in Figure 1.

### 2.1. Motilities and Virulence Factors Production

Motilities allow bacteria to progress through different surfaces and/or tissues and different forms of motilities can be adopted, depending on bacterial species. Briefly, four active motilities have been reported: swimming, swarming, and twitching, that rely on the presence of flagella and/or type IV pili, and gliding that requires a slime production [19]. For instance, the monotrichous-flagellated *P. aeruginosa* (Figure 1A) and peritrichous-flagellated *E. coli* (Figure 1B) have been reported to swim, swarm, and twitch whereas the non-flagellated *S. aureus* (Figure 1C) is able of gliding by the production of slime guns. Additionally, two passive surface translocations, called sliding and darting, have been evoked, particularly for bacteria that lack the required flagella and type IV pili such as *S. aureus* [17]. All these motilities allow invasive bacteria to reach appropriate tissues, depending on their tropisms. Once bacteria recognize the host cell receptors or nutrient-rich surroundings, they will colonize the cell surface and release virulence factors.

The ability of bacteria to produce diffusible as well as non-diffusible virulence factors is important in the development and perpetuation of infection [20]. Diffusible virulence factors include enzymes, toxins, pigments, and surfactants that allow bacteria to cause damage to host cells and despoil their nutrient. Non-diffusible (‘membrane-associated’) virulence factors—including flagella, pili, and capsule lipopolysacharrides and lipoproteins—allow bacteria to adhere to their host tissues but also to counteract and evade immune responses. Particularly, the encapsulation of bacteria inhibits the capacity of macrophages and neutrophils to phagocyte them [21]. Moreover, the production of virulence factors lead to severe host damage, well beyond colonized regions; this is particularly the case of exotoxins that can severely dysregulate critical cellular processes. For instance, (i) enterotoxins produced by enterotoxigenic *E. coli* (ETEC) are a subset of exotoxins that specifically affect the digestive tract and generally result in infectious diarrhea [22]; (ii) the redox properties of pyocyanins permit *P. aeruginosa* to kill competing microbes as well as mammalian cells and to weaken the immune system of infected lungs during cystic fibrosis [23]; (iii) hemolysins and leukotoxins produced by *S. aureus* lyse red and white blood cells, including neutrophils, monocytes, granulocytes, and macrophages which severely undermines innate immune defenses [24].

### 2.2. Formation of Biofilms

The biofilm lifestyle is a bacterial behavior in which organized communities of bacteria are encased in an adhesive and protective matrix of extracellular polymeric substances that hold microbial cells together to a surface; the bacterial biofilms allow colonization and persistence over host surfaces as well as evasion from immune cells phagocytosis. Although the biofilm is not a prerequisite for persistent infection [25], it represents a virulence phenotype significantly protecting bacteria from environmental aggressions.

The biofilm formation is relatively similar from one bacteria species to another, a sequence summarized in four major steps: adhesion, microcolonies development, biofilm maturation, and dispersion [26,27,28]. The extracellular matrix production, initiated at microcolonies development and continued all along the maturation of biofilm, has an adhesin function, allowing bacterial communities to form a rigid and stable structure (Figure 1). These extracellular matrixes are complex environments, essentially composed of water (97%), proteins, nucleic acids, lipids/phospholipids, and exopolysaccharide polymers (EPS), differing according to bacterial species/phenotypes. For instance, (i) *E. coli* produces an EPS mainly represented by cellulose, colanic acid (polymer of glucose, galactose, fucose, and glucuronic acid) and β-1,6-*N*-acetyl-d-glucosamine polymers (PGA) [27]; (ii) *P. aeruginosa* produces at least three types of EPS, mainly alginate (l-guluronic and d-mannuronic acids), Pel (glucose-rich matrix material), and Psl (repeating pentasaccharide units, consisting of d-mannose, l-rhamnose, and d-glucose) that are required for biofilm formation and architecture [29]; and (iii) *S. aureus* mainly produces a glycocalyx primarily composed of teichoic acids and PGA-based exopolysaccharides called “Polysaccharide Intercellular Antigen” (PIA) composed of β-1,6-linked *N*-acetylglucosamine residues and a lower content of non-*N*-acetylated d-glucosaminyl residues that contain phosphate and ester-linked succinate [26].

Interestingly, EPS have been shown to play a major role in the protection properties of biofilm matrix; this is particularly the case for *P. aeruginosa*, in which both components drastically reduce antimicrobials and microbicide penetration, severely reducing the levels of antibiotics in contact with bacterial cells. The formation of biofilm can then be considered as a real mechanism of bacterial resistance to antibiotics [30]. Moreover, the biofilm lifestyle allows the generation of bacterial persister cells, a specific sub-population of bacterial cells that have acquired temporary antibiotic-resistance phenotypes and for which a long-retention effect, or ‘memory effect’, has been demonstrated, even if they return at planktonic lifestyle mode [31].

## 3. Quorum Sensing and Its Entanglement with the Expression of Virulence

QS is a cell-to-cell communication used by many bacteria to coordinate common behaviors and regulate a diverse array of physiological activities through the sensing of cell-population densities. In this process, bacteria produce, release, and detect chemical signal molecules called ‘autoinducers’ (AI) that increase in concentration concomitantly with cell density [32,33,34]. The reach of a minimal threshold stimulatory concentration leads to activation or induction of QS-dependent gene expression among a responding bacterial population. Depending on bacteria species, these processes may include symbiosis, virulence, competence, conjugation, antibiotic production, motility, sporulation, and/or biofilm formation [35]. Basically, there are different types of QS systems based on the nature of AI and their detection mechanism. The best known and/or relevant systems are (i) the LuxI/R signaling systems, primarily exploited by different Gram-negative bacteria such as *Chromobacterium violaceum, Agrobacterium tumefasciens*, and *P. aeruginosa*, based on *N*-acyl homoserine lactones signal molecules (AHLs; also called autoinducer-1, AI-1) and known to promote the production of virulence factors (e.g., violacein, pyocyanin, elastases, and proteases), swarming/twitching motilities, biofilm formation, and other process such as transfer of tumor-inducing plasmid to host [18,36,37]; (ii) the oligopeptide-two-component-type QS system, primarily exploited by Gram-positive bacteria such as *Bacillus cereus, Listeria monocytogenes, Streptococcus mutans, Enterococcus faecalis*, and *S. aureus*, based on autoinducing peptides (AIP) as signal molecules and known to up-regulate the expression of several exoproteins genes (e.g., enterotoxins, listeriolysin O, α-, β-, and γ-hemolysins, as well as leucotoxins) and repress the transcription of particular genes encoding for cell wall-associated proteins (e.g., protein A, coagulase, and fibronectin binding protein) [18,38,39]; (iii) the LuxS/AI-2 signaling system, based on (2R, 4S)-2-methyl-2,3,3,4-tetrahydroxytetrahydrofuran (Autoinducer-2, AI-2), exploited by *Salmonella* spp. and *E. coli* for interspecies communication, and reported to regulate the expression of over 400 genes associated to bacterial adhesion processes, motilities, and toxin production; (iv) the autoinducer-3/epinephrine/norepinephrine (AI-3/E/NE) signaling system based both on pyrazinones (AI-3), primarily exploited by *E. coli* for interkingdom communications [40], and on eukaryotic hormones epinephrine/norepinephrine that are reported to activate a virulence-associated type III secretion system (T3SS), causing characteristic histopathologic ‘attaching and effacing’ lesions on host cells (enterocytes), leading to loss of microvilli and intimate attachment of the bacterium to the host cell surface [41]; and (v) the PhcBSR QS system, based on fatty acids (R)-methyl 3-hydroxymyristate (3-OH MAME) or (R)-methyl 3-hydroxypalmitate (3-OH PAME), employed by *Ralstonia solanacearum* to regulate virulence factors, EPS, and endoglucanase production [42].

For a better understanding, the QS circuitries of the well-studied systems of human pathogen bacteria (*P. aeruginosa, S. aureus*, and *E. coli*) and phytopathogen *R. solanacearum* are succinctly developed in the supplementary materials (Text S1). Overall, the tight modulation of QS systems controlling the production of virulence factor and the attachment process/biofilm formation is a key component of invasion process and bacterial infection success (Figure 1).

## 4. African Plant Extracts with Antivirulence Activities

This section exposes studies reporting African medicinal plants extracts with antivirulence activities. These extracts, that exert their activities against different models of bacteria, are summarized in Table 1.

### 4.1. Activities on Gram-Negative Bacteria

Various South African medicinal plants have been widely investigated for their antivirulence activities against various Gram-negative bacteria:

(i) The hexanic extract from South African *Kigelia africana* (Lam.) Benth. (Fruits, Bignoniaceae) used to treat dysentery, reduces the production of violacein in *C. violaceum* ATCC 12472 by 65% when tested at 660 µg/mL [43]. Additionally, this extract interferes with the QS mechanism in *A. tumefaciens* by affecting the *luxI* synthase gene and the LuxR transcriptional regulator of autoinducers;

(ii) South African plants, used to treat urinary tract infections, have been studied by Baloyi et al. [44] for their effects on the production of violacein in *C. violaceum* ATCC 12472. At a final concentration of 330 µg/mL, the extracts of *Cenchrus ciliaris* L. (bark; methanol; Poaceae), *Cryptocarya latifolia* Sond. (bark; methanol; Lauraceae), *Eucomis autumnalis* (Mill.) Chitt. (bulb; methanol; Aspaagaceae), *Hydnora africana* Thumb. (bark; methanol; Aristolochiaceae), *Hypoxis hemerocallidea* Fisch., C.A.Mey. & Avé-Lall. (corme; dichlorométhane; Hypoxidaceae), *Rhoicissus tridentata* (L.f.) Wild & R.B. Drumm (root; methanol; Vitaceae), *Baccharoides adoensis* (Sch.Bip. ex Walp.) H.Rob. (synonym of *Vernonia adoensis* Sch.Bip. ex Walp.) (bark; aqueous; Asteraceae), *Bauhinia bowkeri* Harv. (root; aqueous; Fabaceae) inhibit the production of violacein in *C. violaceum* ATCC 12472 (from 4 to 43% inhibition);

(iii) Plants from the Myrtaceae family, endemic to South Africa and traditionally used to treat different ailments such as diarrhea, diabetes, reproductive problems, and respiratory diseases, have been investigated for their antibiofilm activity against various Gram-negative bacteria, including *P. aeruginosa* ATCC 27853, *Salmonella* ser. *typhimurium* ATCC 39183, and *E. coli* ATCC 25922 [50]. It has been highlighted that acetone extracts of leaves from different *Syzygium* and *Eugenia* species, particularly *S. legatii* Burtt Davy & Greenway and *E. erythrophylla* Strey, at 1000 µg/mL reduce biofilm formation (from 59–100% inhibition) but were unable to destroy pre-formed biofilms. These extracts exert in planktonic growth condition, a Minimal Inhibitory Concentration (MIC) values ranging from 40 to 310 µg/mL and relatively low cytotoxicity towards kidney epithelial cells (Vero) as concentrations of 140 to 1140 µg/mL inhibit cell viability by 50% (LC_50_); this indicates potentially safe herbal products;

(iv) The ethanolic extract of the leaves of *Lessertia frutescences* (L.) Goldblatt & J.C.Manning (synonym of *Sutherlandia frutescens* (L) R.Br.) (Fabaceae), showed antibiofilm activity (90% inhibition) in *P. aeruginosa* ATCC 35032 and inhibition (>80%) of pyocyanin and LasB elastase [52].

Finally, (v) the methanolic extract of the South African plant *Sclerocarya birrea* (A.Rich.) Hoch. (trunk bark; Anacardiaceae) has been proposed to exhibit anti-QS activity as it inhibits the production of pyoverdine, protease and motility as well as biofilm formation (78% inhibition) in *P. aeruginosa* MTCC 2453 at the concentration of 100 µg/mL [53]. 

Plants from Western Africa, and particularly seven medicinal plants from Burkina Faso, have been also investigated for their impact on *C. violaceum* and *P. aeruginosa* QS mechanism and on *P. aeruginosa* biofilm formation; antivirulence activities of these Burkinabe plants are summarized in Table 2.

These include *Acacia dudgeoni* Craib. ex Holl (Mimosaceae), used in the treatment of diarrhea and childhood dysentery [45,61]; *Balanites aegyptiaca* (L.) Delille (Zygophyllaceae) [48], traditionally used for the treatment of respiratory tract diseases, skin diseases, and wounds; *Crossopteryx febrifuga* (Afzel ex G. Don) Benth. (Rubiaceae) used in the treatment of various infectious diseases such as typhoid fever, respiratory infections, infected wounds, and dental diseases [46]; *Terminalia leiocarpa* (DC.) Baill. (synonym of *Anogeissus leiocarpus* (DC) Guill. et Perr.) (Combretaceae), also used to treat respiratory diseases and wounds [57]; *Terminalia macroptera* Guill. & Perr. (Combretaceae) [48], traditionally used for the treatment of respiratory tract diseases, skin diseases and wounds; *Vachellia seyal* (Delile) P.J.H.Hurter (synonym of *Acacia seyal* Delile) Del (Fabaceae) [47] traditionally used to treat toothache, dysentery, and burns [62] with reported potent antimicrobial activity [63]; and *Zanthoxylum zanthoxyloides* (Lam) Zepern. and Timler (Rutaceae) also used to treat typhoid fever, respiratory infections, infected wounds, and dental diseases [46].

Plants from Eastern Africa have been also investigated for their QQ properties in a *E. coli* model. About 25 medicinal plant extracts (roots, bark, and leaves; ethanol 50% v/v) used in southwestern Kenya to treat gastrointestinal and urinary tract infections were tested against a transformed *E. coli* Top 10 reporter QS strain that expresses green fluorescent protein (GFP) when induced by exogenous AHLs (3-oxo-C6HSL). Interestingly, the extracts of *Vachellia gerrardii* (Benth.) P.J.H.Hurter (synonym of *Acacia gerrardii* Benth) and *Elaeodendron buchananii* (Loes.) Loes. barks, at 1000 µg/mL reduce the reporter GFP expression (up to 50% inhibition) without any effect on the *E. coli* biofilm formation [51]. In the same line, two Ethiopian antimicrobial medicinal plants have been reported to exert anti-QS without affecting bacterial viability; the root methanolic extract of *Albizia schimperiana* Oliv. (Fabaceae) and seed petroleum ether extract of *Justicia schimperiana* (Hochst. ex Nees) T. Anderson (Acanthaceae) at 6500 µg/mL interfere with cell-to-cell communication, most likely by interacting with the 3-oxo-C6-HSL signaling pathway in *E. coli* reporter strain AI1-QQ.1 [49].

For almost 10 years, antivirulence activities of endemic plants from Madagascar have been regularly reported. Indeed, the bark of *D. pervillei* Vakte and *D. trichocarpa* Baker (Fabaceae; hexane extracts tested at 300 µg/mL) inhibit the production of *P. aeruginosa* virulence factors pyocyanin and elastase by 43% and 25%, respectively, and the expression of QS-related (*lasI*, *lasR*, *rhlI*, and *rhlR*) and QS-regulated (*lasB* and *rhlA*) genes [64]. Further investigations on the hexane bark extract of *D. trichocarpa,* traditionally used to treat various ailments—such as laryngitis, diarrhea, and rheumatic pains—led to the generation of active fractions which exert anti-QS and/or antibiofilm activities. Particularly, an active fraction exerts antibiofilm activities at 200 µg/mL (63% inhibition) without affecting bacterial viability or QS mechanism of *P. aeruginosa* PAO1. The inhibition of biofilm formation is probably linked to a reduction in flagellar-dependent motilities (swimming and swarming) as well as in exopolysaccharides production [56]. In Madagascar, a mixture of *Tephrosia purpurea* L. (Fabaceae) and *Buddleja madagascariensis* Lam (Buddlejaceae) macerated in cow dung manure is traditionally used as a phytotreatment against potato crops diseases such as potato leaf spots caused by the phytophatogen *R. solanacearum* [65]. The study of their antibacterial effects demonstrated that the methanolic extracts of both plants, tested at 100 µg/mL, reduce the expression of *P. aeruginosa* PAO1 QS-regulated *lasB* (45% and 52% inhibition, respectively) and *rhlA* (32% and 33% inhibition, respectively) genes; but only *T. purpurea* extracts exhibit antibiofilm activities against *P. aeruginosa* PAO1 and *R. solanacearum* (52% and 30% inhibition, respectively) without affecting bacterial growth [55].

Finally, the dichloromethane extract of *Cordia gilletii* De Wild (Boraginaceae) root barks, medicinal plant from the Democratic Republic of Congo, a plant known for bactericidal activities against pathogenic microorganisms such as *E. coli* [66], was also found to quench the production of pyocyanin, to inhibit the expression of several QS-regulated genes (i.e., *lasB, rhlA, lasI, lasR, rhlI,* and *rhlR;* 35%, 40%, 24%, 25%, 52%, and 23% inhibition, respectively) and to reduce biofilm formation (21% inhibition) in *P. aeruginosa* PAO1 without affecting its viability [54].

### 4.2. Activities on Gram-Positive Bacteria

To date, only South African plants have been investigated for their antivirulence activities against Gram-positive bacteria:

(i) the extracts (concentration, 330 µg/mL) from *Cenchrus ciliaris* L. (bark; methanol; Poaceae), *Eucomis autumnalis* (Mill.) Chitt. (bulb; methanol; Asparagaceae), *Hypoxis hemerocallidea* Fisch., C.A.Mey. & Avé-Lall. (corme; dichloromethane; Hypoxidaceae), *Bacharoides adoensis* (Sch.Bip. ex Walp.) H.Rob. [synonym of *Vernonia adoensis* Sch. Bip. ex Walp.] (bark; water; Asteraceae), which are all used to treat urinary tract infections, inhibit biofilm formation (by 21–38%) in *S. aureus* ATCC 25923 [44];

(ii) the acetone leaves extracts of different *Syzygium* and *Eugenia* species at 1000 µg/mL present antibiofilm activities against *S. aureus* ATCC 29213, *E. faecalis* ATCC 29212, and *B. cereus* ATCC 21366 [43]. Particularly, the extract of *S. gerrardii* (Harv. ex Hook.f.) Burtt Davy had the capacity to reduce biofilm formation in all tested strains (68–100% inhibition) and to destroy pre-formed biofilms (69–100% biofilm dispersion);

(iii) the dichloromethane/methanol extracts of plants traditionally used to treat oral infections, including *Vachellia karroo* (Hyane) Banfi & Galasso (synonym of *Acacia karroo* Hayne) (leaves; Fabaceae), *Erythrina lysistemon* Hutch (bark; Fabaceae), *Spyrostachys africana* Sond. (leaves; Euphorbiaceae), *Tecoma capensis* (Thumb.) Lindl., (leaves; Bignoniaceae) and *Tarchonanthus camphoratus* L. (leaves; Asteraceae), at a concentration of 250 µg/mL, showed antibiofilm activity against *S. mutans* ATCC 25175, known to be responsible for dental caries, with 59%, 68%, 86%, 52%, and 54% inhibition, respectively [59]; 

(iv) when the *L. monocytogenes* ATCC 19111 is grown in the presence of 1000 µg/mL of dichloromethane/methanol leaves extract of *Agathosma betulina* (P.J. Bergius) Pillans, (Rutaceae), and *Aspalathus linearis* (Burm.f.) R. Dahlgren (Fabaceae), plants exclusively found in South Africa, biofilm formation reduction was observed with an inhibition rate of 20% and 75%, respectively [58].

### 4.3. Activities on Gram-Indeterminate Bacteria

Recently, the ethanolic extracts from plants belonging to several South African Medicinal plants have been shown to exert antibiofilm activity in *Mycobacterium smegmatis* MC155, a Gram-indeterminate bacteria [60]. Among the species, *Alectra sessiflora* (Vahl) Kuntze (root; Orobranchaceae), *Eucomis vandermerwei* Verd. (leaves; Asparagaceae), *Euphorbia tirrucalli* L. (Stem; Euphorbiaceae), *Leonotis leonorus* (L.) R.Br (leaves and stems; Lamiaceae), *Salvia africana lutea* L. (leaves and stems; Lamiaceae), *Sphedamnocarpus pruriens* (A. Juss.) Szyszył. (seeds and root; Mapighiaceae), and *Withania somnifera* (L) Dunal (leaves and stems, Solanaceae) reduce biofilm formation by 50% in *M. smegmatis* MC155, at the concentration of 221 µg/mL, 266 µg/mL, 280 μg/mL, 50 µg/mL, 95 µg/mL, 62 µg/mL, and 212 µg/mL, respectively. According to the same study, *Kumara plicatilis* (L.) G.D.Rowley (synonym of *Aloe plicatilis* (L.) Mill.) (roots; Asphodelaceae), *Cassinopsis ilicifolia* (Hochst.) Sleumer (leaves and roots; Icacinaceae), *Dracaena aletriformis* (Haw.) Bos, (leaves; Asparagaceae), *Dracaena draco* (L.) L. (leaves; Asparagaceae), *Faurea saligna* Harv. (leaves; Proteaceae), *Merwilla plumbea* (Lindl.) Speta (leaves; Asparagaceae), *Typha capensis* (Rohrb.) N.E.Br (leaves and roots; Typhaceae) showed reduction of biofilm formation by 50% in *M. smegmatis* MC155 at concentrations > 500 µg/mL. The leaves ethanol extract of *Salvia aurea* L. (synonym of *Salvia africana lutea* L.) (Lamiaceae) and seed ethanol extract of *Sphedamnocarpus pruriens* (A. Juss.) (Malpighiaceae) showed inhibition of *M. tuberculosis* biofilm formation by 50% at concentrations of 95 and 62 μg/mL, respectively. Both showed antimycobacterial activity with MIC values of 31 and 62 μg/mL, respectively, with low cytotoxicity (LC_50_ > 80 µg/mL) towards U937 monocytes cells [60].

## 5. Compounds Isolated from African Plants with Antivirulence Activities

Natural compounds isolated from African plants with antivirulence activities, summarized in Table 3, are exclusively phenolic and terpenoid-based compounds and exert their activity against different bacterial strains.

### 5.1. Activities on Gram-Negative Bacteria

The flavonol epicatechin isolated from *Ficus sansibarica* Warb. (Moraceae), collected in KwaZulu-Natal, South Africa, reduces the adhesion to polystyrene surfaces of Gram-negative *E. coli* ATCC 25922 up to 15% at 3.4 mM [67]. Several active fractions containing flavonoid-like compounds, obtained from the bark of *Combretum albiflorum* (Tul.) Jongkind, (Combretaceae), a Madagascar endemic plant [73], were found to inhibit the production of QS-regulated extracellular virulence factors (violacein in *C. violaceum* CV026 and pyocyanin in *P. aeruginosa* PAO1) [68]. Among these flavonoids, the flavan-3-ol catechin, at 4 mM in *P. aeruginosa* PAO1, had a negative impact on the production of violacein (75% inhibition), pyocyanin (50% inhibition), elastase (30% of inhibition), on biofilm formation (30% inhibition), and on the transcription of several QS-related genes (i.e., *lasI, lasR, rhlI, rhlR, lasB,* and *rhlA*). Intriguingly, synthetic epicatechin reduces the *P. aeruginosa* production of pyocyanin (50% inhibition) and elastase (30% inhibition) without effect on biofilm formation.

The methyl gallate (MG) isolated from the galls (produced on leaves following insect attack) methanol extract of *Guiera senegalensis* J. F. Gmel (Combretaceae), a traditional burkinabe treatment of cough, dysentery, and malaria, has been shown to exert antivirulence activities [69], Methyl gallate presents MIC values of 512 and 64 µg/mL against *P. aeruginosa* PAO1 and *C. violaceum* CV026, respectively, but, at 12.5 µg/mL (67.9 µM), already inhibits the production of pyocyanin (by 65%), and violacein (by 10%). These antivirulence activities are in correlation with the data of Hossain et al. [74] who showed that the production of pyocyanin was inhibited (37–64%) by MG in a concentration-dependent manner (16–256 μg/mL). Moreover, in *P. aeruginosa* PAO1, MG reduces the expression of the AHL synthetases genes (*lasI* and *rhlI*) and the QS regulator genes (*lasR* and *rhlR*) and biofilm formation.

A triterpenoid coumarate ester has been isolated from *Dalbergia trichocarpa* Baker (Malagasy endemic species) bark extract as a major bioactive compound. Indeed, oleanolic aldehyde coumarate (OALC), at 200 µM, inhibits the formation of *P. aeruginosa* PAO1 biofilm (by 44%) and its maintenance as well as the expression of the *las* and *rhl* QS systems [5]. As a consequence, the production of QS-controlled virulence factors, including rhamnolipids, pyocyanin, elastase, and extracellular polysaccharides, as well as twitching and swarming motilities are significantly reduced (75%, 64%, 19%, 44%, 40%, and 52% inhibition, respectively). Additionally, OALC disorganizes established biofilm structure and significantly increases the bactericidal activity of tobramycin against biofilm-encapsulated PAO1 cells. Consistently, in vivo experiments indicated that OALC treatment reduces *P. aeruginosa* pathogenicity in *Caenorhabditis elegans*, a nematode.

The monocyclic diterpenoid cassipourol and the phytosterol β-sitosterol, isolated from *Platostoma rotundifolium* (Briq.) A. J. Paton (Lamiaceae), a Burundian anti-infectious plant [75], inhibit QS-regulated and QS-regulatory genes expression in *las* and *rhl* systems and disrupt the formation of biofilms by *P. aeruginosa* at concentrations down to 12.5 and 50 µM, respectively [70]. Authors also isolated α-amyrin, a biosynthesis precursor of ursolic acid [76], that exerts antibiofilm properties at 50 µM without any effect on QS-regulatory genes expression; this suggests that other ursane and oleane-type triterpenes may exert antibiofilm properties with similar mechanisms of action. The three isolated compounds improve swimming but not twitching motilities which consequently promotes planktonic lifestyle in *P. aeruginosa* PAO1 and dispersal on preformed biofilms. Interestingly, the addition of cassipourol, α-amyrin, and β-sitosterol (100 µM) considerably improved the effectiveness of tobramycin (50 µg/mL = 107 µM) against *P. aeruginosa* PA01 with a drastic reduction in cell viability of biofilm-encapsulated bacteria (89%, 70%, and 76% of bacterial death, respectively, versus 40% in DMSO control treatment).

### 5.2. Activities on Gram-Positive Bacteria

Epicatechin (8000 µg/mL, 3.4 mM) and isovitexin (*apigenin* 6-C-*glucoside*) (200–500 µg/mL) (0.4–1 mM) isolated from *Ficus sansibarica* Warb. subsp. *Sansibarica,* (Moraceae) collected in KwaZulu-Natal, South Africa, were found to decrease the adhesion of methicillin-susceptible *S. aureus* ATCC 29213, indicating the potential of flavonoids as antivirulence agents [68]. However, adhesion of methicillin-resistant *S. aureus* ATCC 43300 was increased in presence of isovitexin and the epicatechin isolated from the South African medicinal plant *V. karroo* does not inhibit biofilm formation by *L. monocytogenes* LMG 21263 [71], suggesting that inhibition of biofilm process by these compounds is strain-specific. Two other compounds (epigallocatechin and β-sitosterol), isolated from *V. karroo*, inhibit *L. monocytogenes* LMG 21263 biofilm formation at the concentration of 500 µg/mL (1.2 mM) [71]. Both present MICs at 31 µg/mL and 62 µg/mL (67 and 149 µM), respectively. Considering these interesting bactericidal and antibiofilm properties, it is regrettable that no investigation on potential synergistic properties with conventional antibiotic has been performed so far; these could strengthen arguments to consider them as potential candidates for drug discovery and development.

Among Malagasy endemic *Dalbergia* species, *D. pervillei* Vatke (Fabaceae) exhibited attenuated gall phenotype when infected by the phytopathogen *Rhodococcus fascians* [72]. Further investigations led to the isolation of a prenylated isoflavanone, perbergin, which has been shown to target *attR* gene expression, encoding a LysR-type transcriptional regulator that plays a key role in regulating the expression of virulence genes of *R. fascians*, notably its transition from an epiphytic to a pathogenic lifestyle [72]. Perbergin inhibited the induction of bacterial virulence *attR* gene expression without any apparent loss of bacterial viability at 0.2 µM but demonstrated strong bactericidal activity against *R. fascians* at 10 µM.

## 6. Discussion

African plants screened so far provide a clear indication that we have a fairly large source of non-microbicidal natural products active on bacteria (Table 1 and Table 3). According to literature, the search for antivirulence activities have been shyly initiated since the last decade; by contrast, there has been a wide research on conventional antimicrobial activities (i.e., bactericidal activity) of African plants over the past 30 years [77,78,79]. Although African plants investigated for antivirulence activities are diverse (largely from Southern and Eastern African regions), very few studies have resulted in the characterization of the active compound(s), suggesting that this investigation is only beginning, which highlights a huge potential for new substances still to be discovered.

Considering that bacterial strategies to invade hosts may subtly differ according to species as well as within species, with different pathovars, it is difficult to claim that a single bacterial virulence target would be effective to undermine a bacterial invasion process. Although QS seems to be located at the crossroads of bacterial virulence expression, there is no universal QS system and cell-to-cell communication is differently exploited by bacterial species during bacterial infection, meaning that antivirulence compounds will generally be strain-specific.

**Figure 2:**
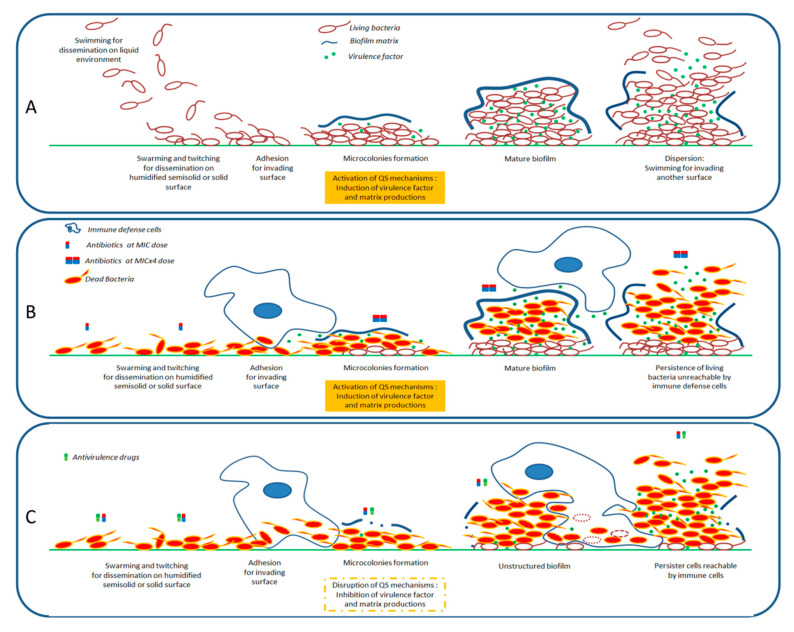
Model of antibiotherapy combined with an antivirulence agent. (**A**) Schematic representation of bacterial invasion processes. (**B**) Scenario presenting the use of antibiotherapy alone and the difficulty of immune defense cells to clear biofilm-encapsulated bacteria even at high doses of antibiotic (e.g., four to 100-fold MICs [82,83]). (**C**) Scenario presenting the simultaneous use of antibiotic and antivirulence agents; antibiotics at MICs kill a majority of bacteria gathered inside an unstructured biofilm which can be easily cleared by immune defense cells.

Millennia of co-evolution between plants and pathogenic bacteria have led to complex defense strategies; invaded plants produce myriads of bactericidal and/or antipathogenic secondary metabolites that target different mechanisms. Although, the present African plants review highlights antivirulence properties, it is interesting to note that some of these plants also harbor bactericidal compounds with effects similar to those of conventional antibiotics. A relevant example is *Platostoma rotundifolium* in which compounds that exert bactericidal activities against *S. aureus* and *E. coli* (ursolic and corrosolic acid) coexist with compounds with antivirulence activities against *P. aeruginosa* (cassipourol, β-sitosterol, and α-amyrin) [70,80]. Presumably, such a strategy allows plants to increase their probability of success in controlling bacterial invasions; this highlights that combined therapies, i.e., the use of two or multiple drugs with different antibacterial mechanisms of action, may represent an optimal strategy to sustainably struggle against bacterial infections, notably when a pathogen agent has not been specifically identified. In an empirical way, this type of strategy is adopted by tradipractices that often propose mixtures of medicinal plants to treat patients [15,81]. Thus, a combination of bactericidal agents and virulence inhibitors is expected to represent an effective therapeutic strategy to efficiently overcome bacterial infections. In this scenario, antivirulence agents would thwart the ability of bacteria to provoke severe infections or to evade immune defenses of the host, which conversely preserves or reinforces the bactericidal effectiveness of antibiotic agents; a rapid clearance of bacteria, including bacterial persister cells, by immune defenses would then be expected at each stage of invasion (Figure 2). Furthermore, in a synergistic approach, the efficiency of antibiotics could be achieved at lower concentrations, minimizing a selective pressure that is known to generate resistant bacteria spreading with collateral damages towards the commensal and symbiotic bacteria of the host.

So far, however, it should be acknowledged that the discovery of QS modulators has not yet led to major therapeutic breakthroughs; also, QS systems do not control the totality of virulence factors expression and the development of anti-QS bacterial resistance cannot be excluded [84]. However, this should not prevent further research in this promising field. Indeed, according to in vitro experiments, the combination of antibiotics and antivirulence (e.g., cassipourol) agents has already demonstrated its potential and about 33 compounds or agents, mainly from synthetic origin, that target virulence factors are under way for preclinical investigations, most of them focusing on *P. aeruginosa, Enterobacteriaceae* spp., and *S. aureus* [85,86,87].

## Figures and Tables

**Table 1:** African medicinal plant extracts with antivirulence activities

Bacterial Model Strains		Plant Family ^1^	Plant Species ^1^	Plant Part and Traditional Usage	Active Extracts (or Fraction)	Bactericidal Effect MIC (µg/mL)	Impact on Bacterial Virulence ^2^				Synergy with Antibiotics	References
							Motility	Virulence Factors Production	Biofilm Formation	QS		
Group of Gram-negative bacteria	*A. tumefaciens*	Bignoniaceae	*Kigelia africana* (Lam.) Benth	Fruit; dysentery, toothaches, malaria, diabetes	Hexane	NC	NC	↘	NC	↘	NC	[43]
	*C. violaceum*	Asteraceae	*Baccharoides adoensis* (Sch. Bip. ex Walp.) H. Rob. [synonym of *Vernonia adoensis* Schi.Bip. ex Walp.]	Bark; urinary tract infections	Distilled water	4000	NC	↔	↘	NC	NC	[44]
	*C. violaceum* ATCC 12472	Aristolochiaceae	*Hydnora africana* Thumb.	Bark; urinary tract infections	Methanol	1000	NC	↘	↘	NC	NC	[44]
		Aspagaceae	*Eucomis autumnalis* (Mill.) Chitt	Bulb; urinary tract infections	Methanol	2000–4000						
		Bignoniaceae	*Kigelia africana* (Lam.) Benth	Fruit; dysentery, toothaches, malaria, diabetes	Hexane	NC	NC	↘	NC	↘	NC	[43]
		Hypoxidaceae	*Hypoxis hemerocallidea* Fish., C.A.Mey. & Avé-Lall.	Corm; urinary tract infections	Dichloromethane	2000	NC	↘	↘	NC	NC	[44]
		Lauraceae	*Cryptocarya latifolia* Sond.	Bark; urinary tract infections	Methanol	2000–4000			↔			
		Poacae	*Cenchrus ciliaris* L.	Leaves; urinary tract infections	Methanol				↘			
		Vitaceae	*Rhoicissus tridentata* (L.f.) Wild & R.B.Drumm	Roots; urinary tract infections	Methanol				↔			
	*C. violaceum* CV026	Combretaceae	*Terminalia leiocarpa* (DC.) Baill. [synonym of Anogeissus *leiocarpus* (DC) Guill. et Perr.]	Stem bark; treat infected burn wounds	Methanol	1250	NC	↘	↔	NC	NC	[45]
			*Terminalia macroptera* Guill. and Perr.	Bark; respiratory tract diseases, skin diseases, and wound			NC	↘	↘	↘	NC	[46]
		Fabaceae	*Vachellia seyal* (Delile) P.J.H.Hurter [synonym of *Acacia seyal* Delile]	Bark; toothache, dysentery, burns	Methanol	NC	NC	↘	NC	NC	NC	[47]
		Mimosaceae	*Acacia dudgeoni* Craib. ex Holland	Bark; diarrhea, childhood dysentery	Methanol	NC	NC	↘	NC	↘	NC	[48]
		Zygophyllaceae	*Balanites aegyptiaca* (L.) Delille.	Galls and stem bark; respiratory tract diseases, skin diseases and wound	Methanol	1250	NC	↘	↘	↔	NC	[46]
	*E. coli*	Acanthaceae	*Justicia schimperiana* (Hochst. ex Nees) T. Anderson	Whole plant; malaria, cough, stomach, asthma	Petroleum ether	NC	NC	NC	NC	↘	NC	[49]
		Fabaceae	*Albizia Schimperiana* Oliv.	Leaves; antifungal	Methanol	NC	NC	NC	NC	↘	NC	[49]
	*E. coli* ATCC 25922	Myrtaceae	*Eugenia erythrophylla* Strey	Leaves; diarrhea, diabetes, reproductive problems, and respiratory conditions	Acetone	80–310	NC	NC	↘	NC	NC	[50]
			*Eugenia umtamvunensis* A.E.van Wyk									
			*Eugenia capensis subsp. zeyheri* (Harv.) F.White [synonym of *Eugenia zeyheri* (Harv.) Harv.]									
			*Syzygium legatii* Burtt Davy & Greenway									
			*Syzygium masukuense* (Baker) R.E.Fr.									
			*Syzygium sp.*									
	*E. coli pBCA9145-jtk2828:: sfGFP*	Celastraceae	*Elaeodendron buchananii* (Loes). Loes.	Roots; gastrointestinal tract, urinary tract, skin, and oral cavity	Ethanol	500	NC	↔	↔	↘	NC	[51]
		Fabaceae	*Vachellia gerrardii* (Benth.) P.J.H.Hurter [synonym of *Acacia gerrardii* Benth]									
	*P. aeruginosa* ATCC 27853	Myrtaceae	*Eugenia erythrophylla* Strey	Leaves; diarrhea, diabetes, reproductive problems, and respiratory conditions	Acetone	80–310	NC	NC	↘	NC	NC	[50]
			*Eugenia umtamvunensis* A.E.van Wyk									
			*Eugenia capensis subsp. zeyheri* (Harv.) F.White [synonym of *Eugenia zeyheri* (Harv.) Harv.]									
			*Syzygium legatii* Burtt Davy & Greenway									
	*P. aeruginosa* ATCC 35032	Fabaceae	*Lessertia frutescens* (L.) Goldblatt & J.C.Manning [synonym of *Sutherlandia frutescens* (L) R. Br.]	Leaves; cancers, fever, diabetes	Ethanol	NC	NC	↘	↘	NC	NC	[52]
	*P. aeruginosa* MTCC 2453	Anacardiaceae	*Sclerocarya birrea* (A.Rich.) Hoch	Stem bark; dysentery, diarrhea	Methanol	NC	↘	↘	↘	NC	NC	[53]
	*P. aeruginosa* PAO1	Baroginaceae	*Cordia gilletii* De Wild	Root barks; malaria, diarrhea, wounds, and skin diseases	Dichloromethane	NC	NC	↘	↘	↘	NC	[54]
		Buddlejaceae	*Buddleja madagascariensis* Lam.	Leaves; potato wilt diseases	Methanol	4000	NC	NC	↘	↘	NC	[55]
		Combretaceae	*Terminalia leiocarpa* (DC.) Baill. [synonym of *Anogeissus leiocarpus* (DC) Guill. et Perr.]	Stem bark; treat infected burn wounds	Methanol	1250	NC	↘	↔	NC	NC	[45]
		Fabaceae	*Vachellia seyal* (Delile) P.J.H.Hurter [synonym of *Acacia seyal* Delile]	Bark; toothache, dysentery, burns	Methanol	NC	NC	↘	↘	NC	NC	[47]
			*Dalbergia trichocarpa* Baker	Bark; laryngitis, diarrhea	Hexane (F1 Fraction)	4000	↘	↘	↘	↔	yes	[56]
			*Tephrosia purpurea* (L.) Pers.	Leaves; potato wilt diseases	Methanol	4000	NC	NC	↘	↔	NC	[55]
		Mimosaceae	*Acacia dudgeoni* Craib. ex Holland	Bark; diarrhea, childhood dysentery	Methanol	NC	NC	↘	↘	NC	NC	[48]
		Rubiaceae	*Crossopteryx febrifuga* (Afzel ex G. Don) Benth	Leaves and stem; typhoid fever, respiratory infections, infected wounds, dental diseases	Methanol	NC	NC	↘	NC	NC	NC	[57]
		Rutaceae	*Zanthoxylum zanthoxyloides* (Lam) Zepern. and Timler	Stem bark; typhoid fever, respiratory infections, infected wounds, dental diseases								
		Zygophyllaceae	*Balanites aegyptiaca* (L.) Delille	Galls and stem bark; respiratory tract diseases, skin diseases and wound	Methanol	625	NC	↘	↘	↔	NC	[46]
	*R. solanacearum*	Fabaceae	*Tephrosia purpurea* (L.) Pers.	Leaves; potato wilt diseases	Methanol	4000	NC	NC	↘	↔	NC	[55]
		Scrophulariaceae	*Buddleja madagascariensis* Lam	Leaves; potato wilt diseases	Methanol	4000	NC	NC	↘	↘	NC	
	*Salmonella* ser. Typhimurium ATCC 39183	Myrtaceae	*Eugenia erythrophylla* Strey	Leaves; diarrhea, diabetes, reproductive problems, and respiratory conditions	Acetone	40–310	NC	NC	↘	NC	NC	[50]
			*Eugenia umtamvunensis* A.E.van Wyk									
			*Eugenia capensis subsp. zeyheri* (Harv.) F.White [synonym of *Eugenia zeyheri* (Harv.) Harv.]									
			*Syzygium legatii* Burtt Davy & Greenway									
			*Syzygium masukuense* (Baker) R.E.Fr.									
			*Syzygium sp.*									
Group of Gram-positive bacteria	*B. cereus* ATCC 21366	Myrtaceae	*Eugenia erythrophylla* Strey	Leaves; diarrhea, diabetes, reproductive problems, and respiratory conditions	Acetone	20–160	NC	NC	↘	NC	NC	[50]
			*Eugenia umtamvunensis* A.E.van Wyk									
			*Eugenia capensis subsp. zeyheri* (Harv.) F.White [synonym of *Eugenia zeyheri* (Harv.) Harv.]									
			*Syzygium legatii* Burtt Davy & Greenway									
			*Syzygium sp.*									
			*Syzygium gerrardii* (Harv. ex Hook.f.) Burtt Davy									
	*E. faecalis* ATCC 29212	Myrtaceae	*Eugenia umtamvunensis* A.E.van Wyk	Leaves; diarrhea, diabetes, reproductive problems, and respiratory conditions	Acetone	40–160	NC	NC	↘	NC	NC	[50]
			*Syzygium masukuense* (Baker) R.E.Fr.									
			*Syzygium legatii* Burtt Davy & Greenway									
			*Syzygium gerrardii* (Harv. ex Hook.f.) Burtt Davy									
	*L. monocytogenes* ATCC 19111	Fabaceae	*Aspalathus linearis* (Burn.f.) R. Dahlgren	Leaves; antioxidant and antifungal activities	Dichloromethane/Methanol	NC	NC	NC	↘	NC	NC	[58]
		Rutaceae	*Agathosma betulina* (P.J. Bergius) Pillans	Dried plant material; urinary tract infectious								
	*S. aureus* ATCC 25923	Aristolochiaceae	*Hydnora africana* Thumb.	Bark; urinary tract infections	Methanol	500–4000	NC	↘	↘	NC	NC	[44]
		Asparagaceae	*Eucomis autumnalis* (Mill.) Chitt	Bulb; urinary tract infections								
		Asteraceae	*Baccharoides adoensis* (Sch.Bip. ex Walp.) H.Rob. [synonym of *Vernonia adoensis* Sch.Bip. ex Walp.]	Bark; urinary tract infections	Distilled water			↔	↘			
		Fabaceae	*Bauhinia bowkeri* Harv.	Roots; urinary tract infections	Water			↘	↔			
		Hypoxidaceae	*Hypoxis hemerocallidea* Fisch., C.A.Mey. & Avé-Lall.	Corm; urinary tract infections	Dichloromethane			↘	↘			
		Lauraceae	*Cryptocarya latifolia* Sond.	Bark; urinary tract infections	Methanol			↘	↔			
	*S. aureus* ATCC 29213	Myrtaceae	*Eugenia erythrophylla* Strey	Leaves; diarrhea, diabetes, reproductive problems, and respiratory conditions	Acetone	40–160	NC	NC	↘	NC	NC	[50]
			*Eugenia umtamvunensis* A.E.van Wyk									
			*Eugenia capensis subsp. zeyheri* (Harv.) F.White [synonym of *Eugenia zeyherii* (Harv.) Harv.]									
			*Syzygium legatii* Burtt Davy & Greenway									
			*Syzygium masukuense* (Baker) R.E.Fr.									
			*Syzygium gerrardii* (Harv. ex Hook.f.) Burtt Davy									
	*S. mutans* ATCC 25175	Asteraceae	*Tarchonanthus camphoratus L.*	Leaves; toothache	Dichloromethane/methanol	500–1000	NC	NC	↘	NC	NC	[59]
		Bignoniaceae	*Tecoma capensis* (Thumb.) Lindl.,	Leaves; rubbed onto bleeding	Dichloromethane/methanol							
		Euphorbiaceae	*Spyrostachys africana* Sond.	Leaves; toothache remedy	Dichloromethane/methanol							
		Fabaceae	*Vachellia karroo* (Hayne) Banfi & Galasso [synonym of *Acacia karroo* Hayne]	Leaves; oral thrush	Dichloromethane/methanol							
		Fabaceae	*Erythrina lysistemon* Hutch.	Bark; toothache	Dichloromethane/methanol							
Group of Gram-indeterminate bacteria	*Mycobacterium smegmatis* MC155 *M.tuberculosis H37Rv ATCC 27264*	Asphodelaceae	*Kumara plicatilis* (L.) G.D.Rowley [synonym of *Aloe plicatilis* (L.) Mill.]	Roots; diarrhea	Ethanol	31–1000	NC	NC	↘	NC	NC	[60]
		Asparagaceae	*Dracaena aletriformis* (Haw.) Bos	Leaves; chest pains								
			*Dracaena draco* (L.) L.	Leaves; fever, respiratory ailments								
			*Eucomis vandermerwei* Verd.	Leaves; anti-inflammatory								
			*Merwilla plumbea* (Lindl.) Speta	Leaves; chest pains and lung infections								
		Euphorbiaceae	*Euphorbia tirrucalli* L.	Stems; cough								
		Icacinaceae	*Cassinopsis ilicifolia* (Hochst.) Sleumer	Leaves and stem; stomach ailments								
		Lamiaceae	*Leonotis leonurus* (L) R.Br.	Leaves and stem; fever, headache, and cough								
			*Salvia aurea* L. [synonym of *Salvia africana lutea* L.]	Leaves and stem; cough, colds, and bronchitis								
		Malpighiaceae	*Sphedamnocarpus pruriens* (A. Juss.) Szyszył	Seeds and roots; snake bites								
		Orobanchaceae	*Alectra sessiliflora* (Vahl) Kuntze	Roots; diarrhea								
		Proteaceae	*Faurea saligna* Harv.	Leaves; diarrhea								
		Solanaceae	*Withania somnifera* (L) Dunal	Leaves and stem; fever and anti-inflammatory								
		Typhaceae	*Typha capensis* (Rohrb.) N.E.Br.	Leaves and roots; diarrhea								

^1^ All plant names and families were verified through the Medicinal Plant Names Services (MPNS) of Kew Royal Botanic Gardens (https://www.kew.org/science/our-science/science-services/medicinal-plant-names-services) and, if not described in, MPNS through the Plant List portal (http://www.theplantlist.org/); ^2^ ↗: Promotion; ↘: Inhibition; ↔: no impact; NC: not communicated.

**Table 2:** Antivirulence activities of Burkinabe medicinal plants against Gram-negative bacteria.

Burkinabe Medicinal Plant	Plant Part	Tested Extract and Concentration	Production of Violacein in *C. violaceum* CV026	Production of Pyocyanin in *P. aeruginosa* PAO ^1^	Production of Elastase in *P. aeruginosa* PAO1	Production of Biofilm in *P. aeruginosa* PAO ^1^	References
*Acacia dudgeoni* Craib. ex Holl.	Stem bark	Methanol 50–400 µg/mL ^2,3^	−25% to −69%	−33% to −66%	NC	−25% to −59%	[61]
*Balanites aegyptiaca* (L.) Delille.	Leafy galls ^1^	Methanol 100 µg/mL ^2^	−10%	−15%	NC	−33%	[48]
	Stem bark	Methanol 100 µg/mL ^2^	−15%	−20%	NC	−20%	
*Crossopteryx febrifuga* (Afzel ex G. Don) Benth	Leave and stem	Methanol 100 µg/mL ^2^	NC	−52%	−48%	NC	[46]
*Terminalia leiocarpa* (DC.) Baill. [synonym of *Anogeissus leiocarpus* (DC) Guill. et Perr.]	Stem bark	Methanol 100 µg/mL ^2^	−50%	−66%	NC	NC	[57]
*Terminalia macroptera* Guill. and Perr.	Stem bark	Methanol 100 µg/mL ^2^	−35%	−50%	NC	−30%	[48]
*Vachellia seyal* (Delile) P.J.H.Hurter [synonym of *Acacia seyal* Delile]	Bark	Methanol 50–800 µg/mL ^2,3^	−25% to −97%	−22% to −86%	−8% to −56%	At 800 µg/mL: −69%	[47]
*Zanthoxylum zanthoxyloides* (Lam) Zepern. and Timler	Stem bark	Methanol 100 µg/mL ^2^	NC	−28%	−15%	NC	[46]

^1^ produced on leaves, following insect attack; ^2^ sub-inhibitory concentrations; ^3^ sub-inhibitory concentrations: <800 µg/mL.

**Table 3:** Natural compounds with antivirulence activities isolated from African medicinal plants.

Bacterial Model Strains		Plant Family ^1^	Plant Species ^1^	Compound	Chemical Structure	Plant Part and Traditional Usage	Bactericidal Effect MIC (µg/mL)	Impact on Bacterial Virulence ^2^				Synergy with Antibiotics	References
								Motility	Virulence Factors Production	Biofilm Formation	QS		
Group of Gram-negative bacteria	*E. coli* ATCC 25922	Moraceae	*Ficus sansibarica* Warb.	Epicatechin	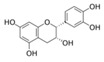	Leaves and fruits; wound, dysentery, diarrhea, tuberculosis	8000	NC	NC	↘	NC	NC	[67]
	*C. violaceum* CV026	Combretaceae	*Combretum albiflorum* (Tul.) Jongkind	Catechin	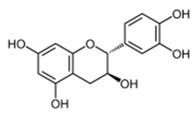	Bark; bacterial infection, fever, pneumonia	2000	NC	↘	NC	↘	NC	[68]
		Combretaceae	*Guiera senegalensis* J. F. Gmel	Methyl Gallate	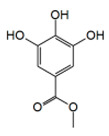	Bark; cough, dysentery, malaria	64	NC	↘	NC	↘	NC	[69]
	*P. aeruginosa* PAO1						512	↘	↘	↘	↘	NC	
		Combretaceae	*Combretum albiflorum* (Tul.) Jongkind	Catechin	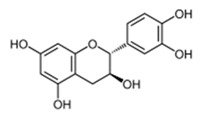	Bark; bacterial infection, fever, pneumonia	2000	NC	↘	↘	↘	NC	[68]
		Fabaceae	*Dalbergia trichocarpa* Baker	Oleanolic aldehyde coumarate	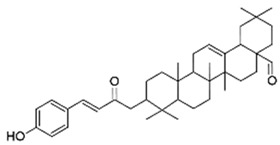	Bark; laryngitis, diarrhea	4000	↘	↘	↘	↘	yes	[5]
		Lamiaceae	*Platostoma rotundifolium* (Briq.) A. J. Paton	β-sitosterol	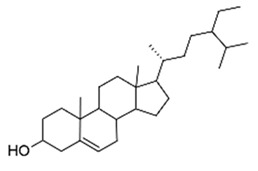	Aerial part; bacterial diseases	4000	↗	↘	↘	↘	yes	[70]
				Cassipourol	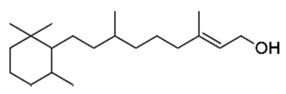		4000	↗	↘	↘	↘	yes	
				α-amyrin	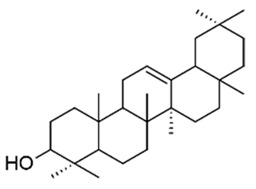		4000	↗	↘	↘	↔	yes	
Group of Gram-positive bacteria	*L. monocytogenes* LMG 21263	Fabaceae	*Vachellia karroo (Hyane) Banfi & Galasso [synonym of Acacia karroo Hayne]*	Epigallocatechin	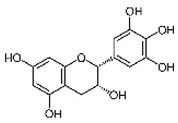	Leaves; dysentery, diarrhea,	31	NC	NC	↘	NC	NC	[71]
				β-sitosterol	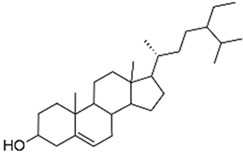		62	NC	NC	↘	NC	NC	
	*R. fascians* D188		*Dalbergia pervillei* Vatke	Perbergin	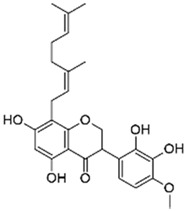	Bark; unknown	5	NC	↘	NC	NC	NC	[72]
	*S. aureus* ATCC 29213	Moraceae	*Ficus sansibarica* Warb.	Isovitexin (apigenin 6C glycoside)	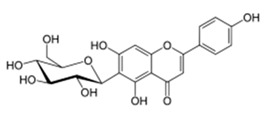	Leaves and fruits; dysentery, diarrhea, wound, tuberculosis	1600	NC	NC	↘	NC	NC	[68]
				Epicatechin	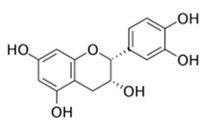		8000	NC	NC	↘	NC	NC	

^1^ All plant names and families were verified through the Medicinal Plant Names Services (MPNS) of Kew Royal Botanic Gardens (https://www.kew.org/science/our-science/science-services/medicinal-plant-names-services) and, if not described in, MPNS through the Plant List portal (http://www.theplantlist.org/); ^2^ ↗: Promotion; ↘: Inhibition; ↔: no impact; NC: not communicated.

## Supplementary Materials

The following are available online at https://www.mdpi.com/2079-6382/9/11/830/s1, Text S1: The QS circuitries of *Pseudomonas aeruginosa, Staphylococcus aureus, Escherichia coli*, and *Ralstonia solanacearum*.

## Abbreviation

AHL	acyl homoserine lactone
AI	autoinducer
EPS	exopolysaccharide polymers
ETEC	enterotoxigenic *E. coli*
HSL	homoserine lactone
MIC	minimal inhibition concentration
QQ	quorum quenching
QS	quorum sensing
