# Supplementary material for: African Plant-Based Natural Products with Antivirulence Activities to the Rescue of Antibiotics
Source: Antibiotics (Basel). 2020 Nov 19;9(11):830. doi: 10.3390/antibiotics9110830 (PMC7699609; doi:10.3390/antibiotics9110830)
Supplement: Supplementary file 1 [file antibiotics-09-00830-s001.pdf]

antibiotics

MDPI

Supplementary materials

Text S1. The QS circuitries of *Pseudomonas aeruginosa, Staphylococcus aureus, Escherichia coli* and *Ralstonia solanacearum.*

The opportunistic human and plant pathogen *P. aeruginosa,* known to be responsible for both acute and chronic infections, possesses three QS systems that have been clearly demonstrated to regulate virulence expression, i.e. two LuxI/R type systems (the *las* and *rhl* systems), based on acyl-homoserine lactones (AHLs), and the PQS (Pseudomonas Quinolone Signal) system based on 2-alkyl-4-quinolones (Figure S1). Beyond this coordination, QS mechanisms are also implicated in swarming and twitching motilities and biofilm formation [1], all of which are important properties for the success and perpetuation of bacterial infection.

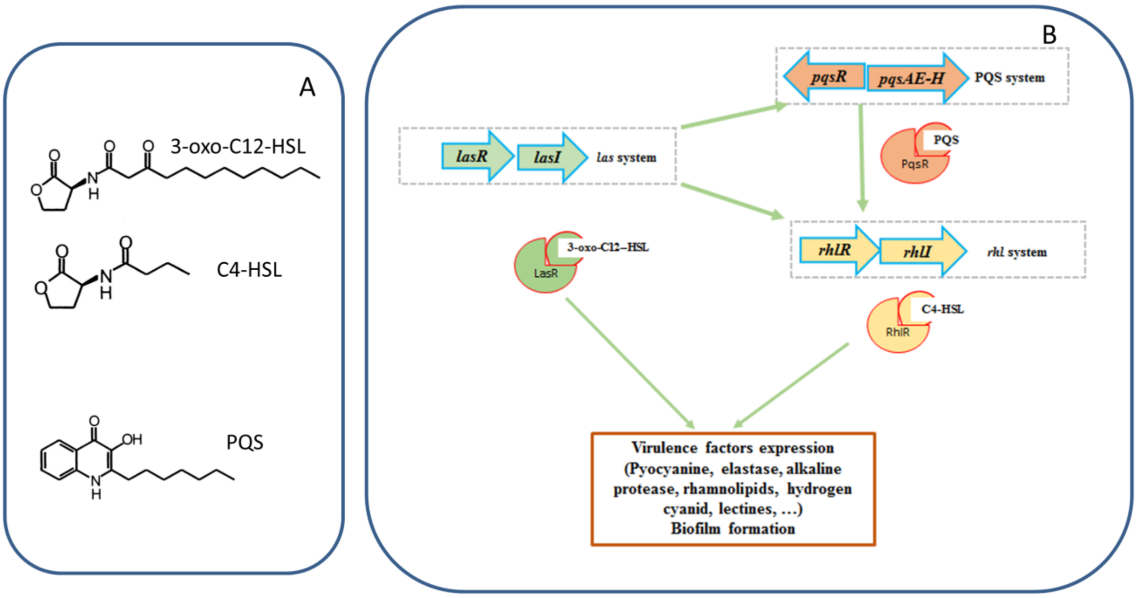

Figure S1. QS mechanisms in *P. aeruginosa.* (A) Main autoinducers used by *P. aeruginosa.* (B) Systems involved in *P. aeruginosa* QS circuitry: *P. aeruginosa* possesses two recognized LuxI/R-type QS systems (*las and rhl*) that drive the production (by the synthetases LasI and RhlI) and the detection (by the receptor LasR and RhlR) of the autoinducer signaling molecules N-(3-oxododecanoyl)-L-homoserine lactone (3-oxo-C12-HSL) and *N*-butanoyl-L-homoserine lactone (C4-HSL), respectively. Additionally, a third non-AHL QS system (Pseudomonas Quinolone Signal), based on 2-alkyl-4-quinolones, synthetized by the PqsABCDE-H operon and detected by the receptor PqsR, is interposed between the two main systems. The complex transcription factors formed by AIs and their correspondent receptors upregulate the expression of QS-regulated genes, leading to the release of virulence factors, such as pyocyanin, lectins, elastase lasB, alkaline protease, exotoxin A and rhamnolipids.

S. aureus, the most common human commensal Gram-positive, is also responsible of pneumonia, endocarditis, osteomyelitis, wound infections, and other complications. This bacterium utilizes primarily the accessory gene regulator (*agr*) system that up-regulates the expression of several exoproteins (*e.g.*, α-, β-, γ-hemolysin, and leucotoxins) and represses the transcription of some cell wall-associated proteins (e.g., protein A, coagulase, and fibronectin binding protein) [2]. The *agr* QS system, briefly detailed in Figure S2 is based on a two-components system encoded by *agrBDCA* operon.

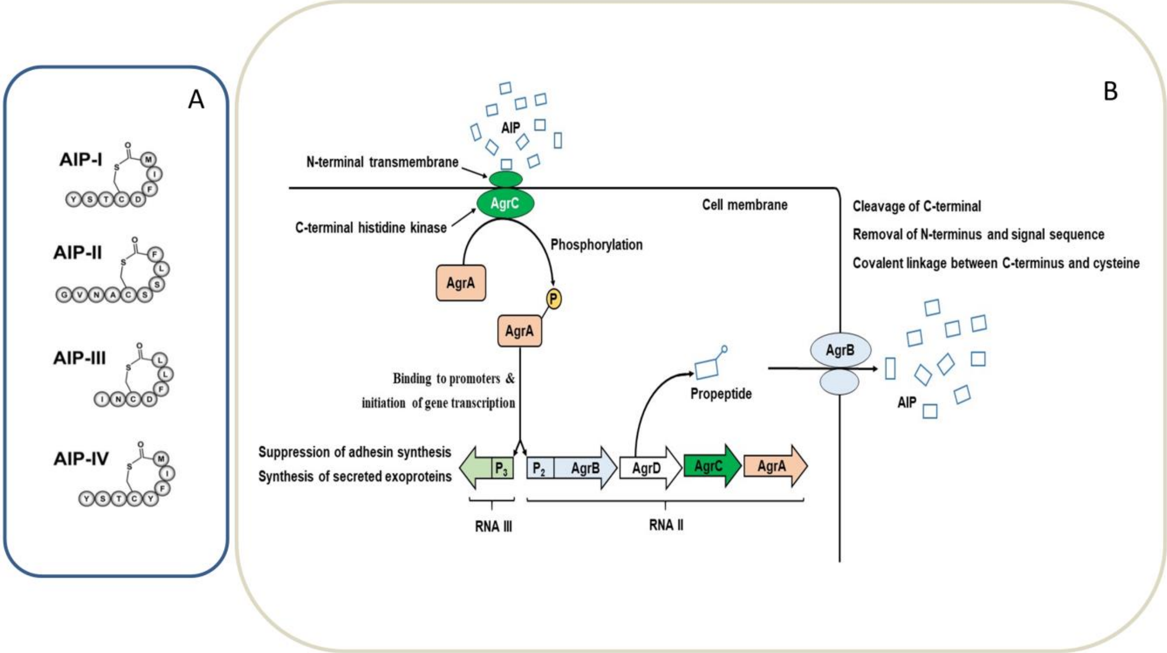

Figure S2. QS mechanisms in *S. aureus.* (A) Main autoinducers used by *S. aureus.* (B) Systems involved in *S. aureus* QS circuitry: The agr QS system is based on a two-components system encoded by the *agrBDCA* operon. AgrC is a transmembrane protein that can bind AIPs (encoded by the *agrD* gene, modified and secreted through the membrane-bound AgrB). Once an AIP binds to ArgC, ArgC transfers a phosphate to the response regulator ArgA, which primarily activates the promoter P2 that drives the transcription of RNAII, the *arg* operon, for autoregulation and activates the promoter P3, leading to the transcription of the RNAIII which in turn leads to the repressed expression of cell adhesion factors and induced expression of secreted factors, including hemolysins, and Panton–Valentine leucocidin [3,4]. Interestingly, *S. aureus* strains possess one of four different classes of Agr systems, each recognizing a unique AIP structure (referred to as Agr-I, Agr-II, Agr-III, and Agr-IV; similarly, their cognate signals are termed AIP-I through AIP-IV).

E. coli presents over 250 serotypes of which some are harmless but commensal and other dreadful intra- or extraintestinal pathogens. The LuxS/AI-2 signaling system is used by *E. coli* to regulate the expression of over 400 genes associated to bacterial adhesion processes, motilities and toxins production whereas the AI-3 and epinephrine/norepinephrine signaling system is particularly used to activate virulence-associated type III secretion systems (T3SS). Both systems are briefly detailed in Figure S3. Interestingly, these QS systems seem to exhibit dual properties in virulence expression of *E. coli*. For instance, in ETEC strains, AI-2 might be a negative regulator of stable-heat (ST) enterotoxin as overexpression of the *luxS* gene reduces the ability of ETEC to induce enterocytes death [5] whereas the labile-heat (LT) enterotoxin seems to be induced by the AI-3/E/NE signaling system [6]. In Enterohemorrhagic *E. coli* O157:H7 (EHEC) and Enteropathogenic *E. coli* (EPEC), both *luxS* type and AI-3/E/NE QS systems activate the transcription of genes localized on the locus enterocyte effacement (LEE) [7,8], genes that encode the T3SS, causing characteristic histopathologic *"attaching and effacing"* lesions on host cells (enterocytes), leading to loss of microvilli and intimate attachment of the bacterium to the host cell surface [9].

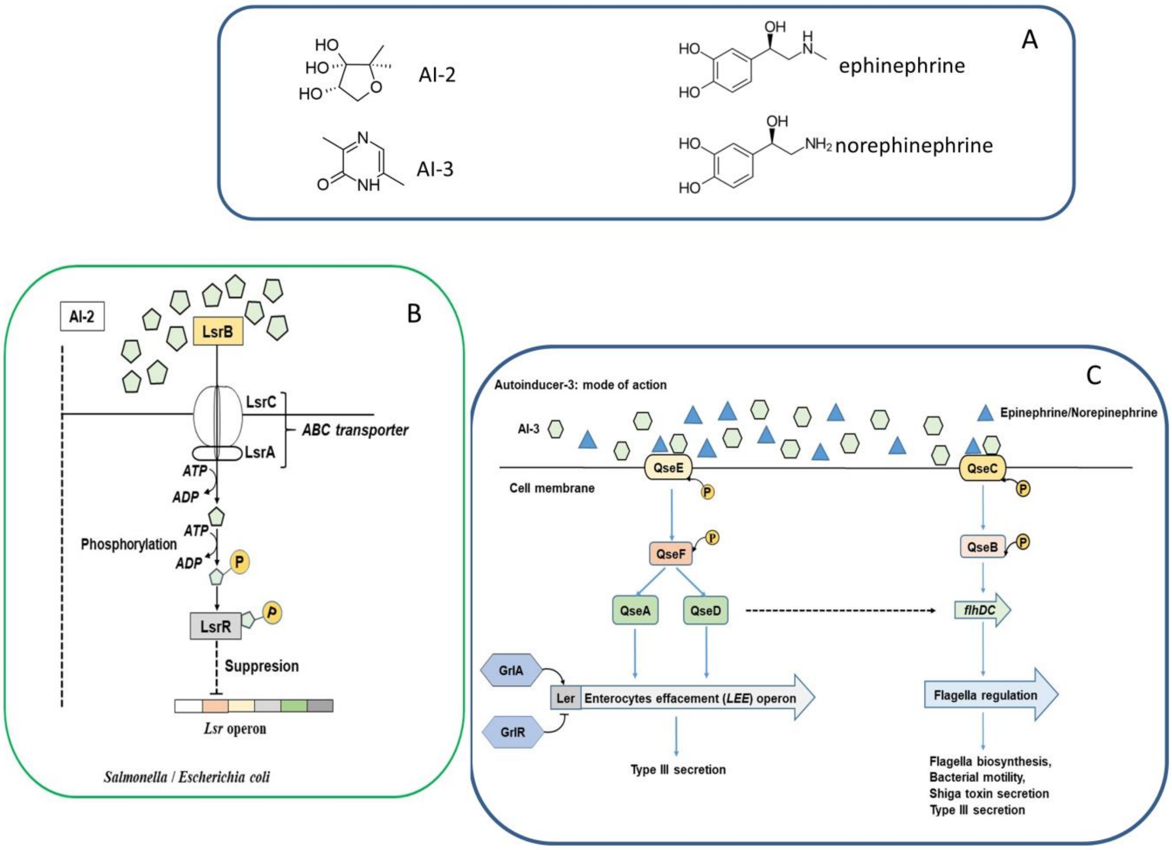

Figure S3. QS mechanisms in *E. coli.* (A) Main Autoinducers used by *E. coli.* (B) The LuxS/AI-2 QS system. In *E. coli* the AI-2 receptor is the LsrB periplasmic protein. Upon binding LsrB, AI-2 is transported inside the cell via the Lsr ABC transport system. AI-2 is then phosphorylated by LsrK, and presumed to interact with LsrR. LsrR is a transcriptional repressor of the lsr operon. Upon complexing of LsR to AI-2, LsrR no longer represses the transcription of *lsr* . Genes in the *lsr* operon are responsible for the AI-2 uptake and modification mechanisms (negative regulator of stable-heat (ST) enterotoxin). (C) The AI-3/ epinephrine/norepinephrine signaling system. AI-3 (produced by the normal gastro-intestinal flora) and epinephrine/norepinephrine (produced by the host) are sensed by Enterohemorrhagic *E. coli* through membrane-bound receptor complexes QseEF and QseBC, respectively. The former complex upregulates the LEE operon, resulting in type III secretion; the latter complex activates the flagella regulon to synthesize flagellae and labile-heat (LT) enterotoxin production.

The phytopathogen *Ralstonia solanacearum* is known to cause bacterial wilt, also known as potato brown rot. This Gram-negative bacterium employ the QS system composed of the phcBSR operon to produce (R)-methyl 3-hydroxymyristate (3-OH-MAME) or (R)-methyl 3-hydroxypalmitate (3-OH-PAME) as signal molecules (Figure S4). In *R. solanacearum,* PhcA represses the production of virulence factors and activates the production of EPS and endoglucanase. At low concentrations of 3-OH-PAME/3-OH-MAME, PhcR is phosphorylated by PhcS and represses PhcA, leading to an increased production of virulence factors. At high concentrations of 3-OH PAME, phosphorylation of PhcR is reduced and PhcA is not repressed, leading to an increased production of EPS and endoglucanase [10].

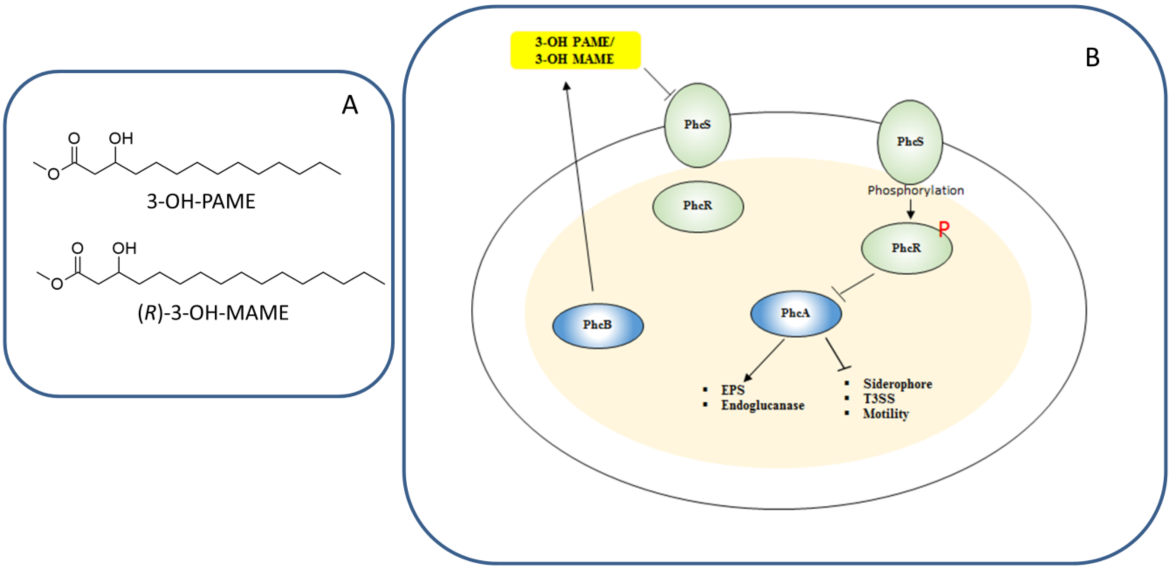

Figure S4. QS mechanisms in *Ralstonia solanacearum.* (A) Main Autoinducers used by *Ralstonia solanacearum.* (B) The PhcBSR quorum-sensing system. PhcA downregulates the production of virulence factors, motilities and T3SS by upregulating EPS and endoglucanase production. The activity of PhcA is regulated by accumulated QS signal 3-hydroxypalmitic acid methyl ester (3-OH-PAME) or (R)-methyl 3-hydroxymyristate (3-OH-MAME) which are encoded by phcB. The two-components system, encoded by *phcS* and *phcR* in the same operon as *phcB*, is involved in detection and response to the QS signal 3-OH-PAME and 3-OH-MAME. Phosphorylated PhcR represses PhcA, leading to an increase of virulence factors production. High concentrations of 3-OH-PAME/3-OH-MAME, detected by PhcS, hinder the phosphorylation of PhcR, that is then unable to repress PhcA, leading to the active production of EPS and endoglucanase.

Reference

1. Jimenez, P.N.; Koch, G.; Thompson, J.A.; Xavier, K.B.; Cool, R.H.; Quax, W.J. The Multiple Signaling Systems Regulating Virulence in *Pseudomonas aeruginosa*. *Microbiol. Mol. Biol. Rev*. **2012**, *76*, 46–65.
2. Bronner, S.; Monteil, H.; Prévost, G. Regulation of Virulence Determinants in *Staphylococcus aureus*: Complexity and Applications. *FEMS Microbiol. Rev*. **2004**, *28*, 183–200.
3. Rutherford, S.T.; Bassler, B.L. Bacterial Quorum Sensing: Its Role in Virulence and Possibilities for Its Control. *Cold Spring Harb. Perspect. Med*. **2012**, *2*, 1–26.
4. Oogai, Y.; Matsuo, M.; Hashimoto, M.; Kato, F.; Sugai, M.; Komatsuzawa, H. Expression of Virulence Factors by *Staphylococcus aureus* Grown in Serum. *Appl. Environ. Microbiol*. **2011**, *77*, 8097–8105.
5. Zhu, J.; Yin, X.; Yu, H.; Zhao, L.; Sabour, P.; Gong, J. Involvement of Quorum Sensing and Heat-Stable Enterotoxin a in Cell Damage Caused by a Porcine Enterotoxigenic *Escherichia coli* Strain. *Infect. Immun.* **2011**, *79*, 1688–1695.
6. Sturbelle, R.T.; da Costa de Avila, L.F.; Roos, T.B.; Borchardt, J.L.; dos Santos da Conceicao, R.d.C.; Dellagostin, O.A.; Leite, F.P.L. The Role of Quorum Sensing in *Escherichia coli* (ETEC) Virulence Factors. *Vet. Microbiol*. **2015**, *180*, 245–252.
7. Sperandio, V.; Mellies, J.L.; Nguyen, W.; Shin, S.; Kaper, J.B. Quorum Sensing Controls Expression of the Type III Secretion Gene Transcription and Protein Secretion in Enterohemorrhagic and Enteropathogenic *Escherichia coli*. *Proc. Natl. Acad. Sci. USA* **1999**, *96*, 15196–15201.
8. Asad, S.; Opal, S.M. Bench-to-Bedside Review: Quorum Sensing and the Role of Cell-to-Cell Communication during Invasive Bacterial Infection. *J. Crit. Care* **2008**, *12*, 1–11.
9. Roussel, C.; Cordonnier, C.; Livrelli, V.; Van de Wiele, T.; Blanquet-Diot, S. Enterotoxigenic and Enterohemorrhagic *Escherichia coli*: Survival and Modulation of Virulence in the Human Gastrointestinal Tract. In *Escherichia coli—Recent Advances on Physiology, Pathogenesis and Biotechnological Applications*; Intechopen: London, UK, 2017; pp. 3–24.

10. Li, P.; Yin, W.; Yan, J.; Chen, Y.; Fu, S.; Song, S.; Zhou, J.; Lyu, M.; Deng, Y.; Zhang, L. Modulation of Inter-Kindom Communication by PhcBSR Quorum Sensing System in *Ralstonia solanacearum* Phynotype I Strain GMI1000. *Front. Microbiol*. **2017**, *8*, 1172.
